# Alterations and mechanistic insights of gut microbiota and its metabolites in type 2 diabetes mellitus and Alzheimer's disease

**DOI:** 10.1002/imo2.70020

**Published:** 2025-05-11

**Authors:** Guangyi Xu, Yu An, Yage Du, Zhaoming Cao, Jie Zheng, Jingya Wang, Tingyi Li, Xingen Lei, Yanhui Lu

**Affiliations:** ^1^ School of Nursing Peking University Beijing China; ^2^ Medical Research Center Beijing Institute of Respiratory Medicine and Beijing Chao‐Yang Hospital, Capital Medical University Beijing China; ^3^ Department of Biostatistics School of Public Health, Columbia University New York New York USA; ^4^ Department of Animal Science Cornell University Ithaca New York USA

**Keywords:** Alzheimer's disease, gut microbiota, metabolites, type 2 diabetes mellitus

## Abstract

Epidemiological studies suggest a link between type 2 diabetes mellitus (T2DM) and Alzheimer's disease (AD), possibly due to gut microbiota dysbiosis, although the exact mechanisms are unclear. This narrative review uniquely addresses how gut microbiota‐derived metabolites mediate overlapping pathologies of insulin resistance, neuroinflammation, and amyloidogenesis in T2DM and AD, proposing a framework for dual therapeutic targeting. This narrative review provides an in‐depth examination of the roles and mechanisms of gut microbiota and their metabolites in the context of T2DM and AD. This study indicates that gut microbiota dysbiosis significantly impacts the pathogenesis and progression of both diseases by modulating metabolic pathways, immune functions, and inflammatory responses. Key bacteria, such as *Akkermansia muciniphila* (which releases outer membrane vesicles), *Lactobacillus*, and *Bifidobacterium*, as well as their metabolites like short‐chain fatty acids (SCFAs), bile acids (BAs), lipopolysaccharide (LPS), vitamins, and Trimethylamine N‐oxide (TMAO) regulate T2DM and AD through complex mechanisms. Multiple signaling pathways, including G‐protein coupled receptor 41/43 (GPR41/43), phosphoinositide 3‐kinase (PI3K)/protein kinase B (Akt), Toll‐like receptor 4 (TLR4)/nuclear factor kappa‐light‐chain‐enhancer of activated B cells (NF‐κB), and endoplasmic reticulum (ER) stress‐mediated pathways, are also involved. These findings offer insights into the pathogenesis and potential targeted therapies for T2DM and AD.

## Highlights

- This narrative review uniquely addresses how gut microbiota‐derived metabolites mediate overlapping pathologies of insulin resistance, neuroinflammation, and amyloidogenesis in type 2 diabetes mellitus (T2DM) and Alzheimer's disease (AD), proposing a framework for dual therapeutic targeting.
- This study indicates that the key bacteria, such as *Akkermansia muciniphila* (which releases outer membrane vesicles), *Lactobacillus*, and *Bifidobacterium*, as well as their metabolites like short‐chain fatty acids (SCFAs), bile acids (BAs), lipopolysaccharide (LPS), vitamins, and Trimethylamine N‐oxide (TMAO) regulate T2DM and AD through complex mechanisms.
- These include Toll‐like receptor 4 (TLR4)/nuclear factor kappa‐light‐chain‐enhancer of activated B cells (NF‐κB)‐driven neuroinflammation, phosphoinositide 3‐kinase (PI3K)/protein kinase B (Akt)‐mediated insulin resistance, and microbial amyloid cross‐seeding, which collectively bridge the two diseases. Multiple signaling pathways, such as G‐protein coupled receptor 41/43 (GPR41/43), PI3K/Akt, TLR4/NF‐κB, and endoplasmic reticulum (ER) stress‐mediated pathways, are critically involved in these processes.

## Graphical abstract

This narrative review uniquely addresses how gut microbiota‐derived metabolites mediate overlapping pathologies of insulin resistance, neuroinflammation, and amyloidogenesis in type 2 diabetes mellitus (T2DM) and Alzheimer's disease (AD), proposing a framework for dual therapeutic targeting. This study indicates that the key bacteria, such as *Akkermansia muciniphila* (which releases outer membrane vesicles), *Lactobacillus*, and *Bifidobacterium*, as well as their metabolites like short‐chain fatty acids (SCFAs), bile acids (BAs), lipopolysaccharide (LPS), and Trimethylamine N‐oxide (TMAO) regulate T2DM and AD through complex mechanisms. These include Toll‐like receptor 4 (TLR4)/activated B cells (NF‐κB)‐driven neuroinflammation, phosphoinositide 3‐kinase (PI3K)/protein kinase B (Akt)‐mediated insulin resistance, and microbial amyloid cross‐seeding, which collectively bridge the two diseases. Multiple signaling pathways, such as G‐protein coupled receptor 41/43 (GPR41/43), PI3K/Akt, TLR4/NF‐κB, and endoplasmic reticulum (ER) stress‐mediated pathways, are critically involved in these processes.

## 1 INTRODUCTION

The intestinal microorganisms, also referred to as the gut microbiota, are currently recognized as a complex organ comprised of approximately 500–1000 species of bacteria, with a total number estimated to be approximately 10^14^, exceeding the total number of human cells by more than tenfold [1, 2]. The gut microbiota resides within the gut of animals, where it and its metabolites engage in vital biological processes such as nutrition and metabolism [3], immune regulation [4], antimicrobial defense [5], and the preservation of gut health [6], etc. When intestinal homeostasis changes, the resulting intestinal dysbacteriosis poses a threat to the body's health [7, 8]. Research indicated that intestinal dysbacteriosis is correlated with metabolic diseases such as diabetes, as well as neurodegenerative diseases like Alzheimer's disease (AD) [9, 10, 11].

Type 2 diabetes mellitus (T2DM) constitutes a persistent metabolic aberration distinguished by the presence of insulin resistance (IR) and elevated blood glucose levels [12]. According to statistical data, over 10.5% of adults worldwide currently suffer from diabetes, with health expenditures related to diabetes exceeding $9660 billion and projected to reach $10,540 billion by 2045, posing a significant burden on public health systems [13]. The etiology of T2DM encompasses genetic factors that affect insulin secretion and IR, as well as environmental factors related to unhealthy lifestyles such as smoking, unhealthy diet, and inadequate physical inactivity [14]. Among these factors, unhealthy dietary habits may exert adverse effects on the functionality and composition of the gut microbiota [15, 16]. The consequent disruption of the gut microbiota, arising from these adverse effects, may give rise to intestinal permeability and the unregulated influx of antigens into the circulatory system, ultimately eliciting immune responses, inflammatory reactions, and metabolic derangements [17, 18]. Ultimately, these disruptions may ultimately lead to the impairment of pancreatic β‐cells and the manifestation of diabetes [19, 20, 21].

AD, representing the most prevalent form of dementia, constitutes 60%–80% of all recorded cases [22]. According to epidemiological surveys, the current prevalence rate of AD is estimated to be 760.5 cases per 100,000 residents [23]. Moreover, over the past decade, the number of deaths caused by AD has increased by 145%, rendering it among the foremost contributors to mortality worldwide [24, 25]. The primary clinical manifestations of AD encompass progressive memory impairment, decline in cognitive functions, and behavioral alterations. Pathologically, it is distinguished by the accumulation of amyloid‐beta (Aβ) peptides in both diffuse and neuritic extracellular amyloid deposits (often encircled by dystrophic neurites), alongside the intracellular formation of neurofibrillary tangles (NFTs) induced by phosphorylated Tau (p‐Tau) proteins. These pathological alterations are frequently concomitant with proliferative reactive microglia and the depletion of neurons, white matter, and synaptic connections [26]. Studies have shown that these substantial pathological changes in AD can be regulated by the gut microbiota and their metabolites, particularly highlighting the role of the microbiota‐gut‐brain axis in this process [27, 28]. Epidemiological evidence indicates a robust correlation between T2DM and an elevated risk of AD, despite the incomplete elucidation of the underlying mechanisms [29]. Research has validated that intestinal dysbiosis plays a contributory role in the progression of T2DM and AD [30]. Furthermore, research has demonstrated that metabolites linked to T2DM, including branched‐chain amino acids, may elevate the risk of developing AD [31]. In addition, AD is increasingly termed “Type 3 Diabetes.” However, the molecular interplay between gut microbiota dysbiosis, metabolic dysfunction, and neurodegeneration remains unresolved. This review defines the research problem of identifying shared microbial–metabolic pathways that drive both diseases, offering insights into comorbidity mechanisms.

## 2 THE ROLES OF GUT MICROBIOTA AND ITS METABOLITES ON T2DM AND AD

In recent years, numerous studies [32, 33, 34, 35] have explored the individual impacts of gut microbiota and their metabolites on both T2DM and AD. Nevertheless, in the present epoch where AD is designated as type 3 diabetes, the intricate tripartite interplay among gut microbiota, their metabolites, T2DM, and AD warrants further clarification. Herein, we present a comprehensive overview, highlighting both the shared and distinct impacts of gut microbiota and their metabolites on T2DM and AD.

### The common roles of gut microbiota and metabolites on T2DM and AD

Numerous studies have revealed that during the onset and progression of both T2DM and AD, a multitude of gut microbiota and associated metabolites play pivotal roles in both disorders. Table 1 provides an overview of the modifications in gut microbiota composition and associated metabolites that have been documented in T2DM and AD. The gut microbiota and their metabolites exert common effects on both T2DM and AD. Significantly, disruptions in cerebral glucose utilization, energy metabolism, and insulin signaling have given rise to the notion of AD being regarded as “brain diabetes” or type 3 diabetes [70, 71]. This shift in perspective emphasizes the subtle yet profound connections between T2DM and AD.

**TABLE 1:** Alterations in gut microbiota and associated metabolites in T2DM and AD.

Diseases	Gut microbiota ↑	Gut microbiota ↓	Alterations in metabolites	References
T2DM patients	‐	*Butyricicoccus, Clostridium leptum* group	Succinate **↑** Butyrate, Acetate, BHB**↓**	[36]
	*Bacteroides caccae, Clostridium hathewayi*, *Clostridium ramosum, Clostridium symbiosum*, *Escherichia coli, A. muciniphila*	‐	Butyrate **↓**	[37, 38]
	*Dorea*	*Bifidobacterium, Akkermansia*	Butyrate **↓**	[39]
	*Clostridium bolteae*	*Alistipes, Pseudoflavonifractor, Oscillibacter*	Butyrate **↓**	[40]
	‐	*Subdoligranulum, Clostridium*, *Faecalibacterium* genera	Butyrate **↓**	[36, 38, 40, 41]
	*Enterobacter*	*Clostridium*	Butyric acid **↓**	[42, 43, 44]
	‐	*Roseburia, Eubacterium, Ruminococcus*, *Bacteroides* genera, *Lachnospira*	SCFAs **↓**	[36, 38, 45, 46, 47]
	*Eggerthella lenta*	‐	Imidazopropionic acid **↑** Butyrate **↓**	[37, 40, 48]
	*Streptococcus mutans*	‐	Imidazopropionic acid **↑**	[40, 48]
	*Prevotella copri, Bacteroides vulgatus*	‐	BCAA**↑**	[49]
	*Desulfovibrio sp. 3_1_syn3*	‐	Sulfate reduction **↑**	[37]
	*Escherichia‐Shigella*, *Enterococcus genus, Bifidobacterium*, *Lactobacillus, Megasphaera*	*Blautia, Prevotella*	‐	[38]
	*Bacteroides* genera	‐	‐	[36, 44]
T2DM mice	*Akkermansia*, *Lachnospiraceae*_NK4A136_group	‐	Carbohydrates (such as maltose, galactose, glucose, mannose, and lactulose), AAs (such as alanine, leucine, norvaline, and arginine) **↑**	[50, 51]
	*Parabacteroides*	‐	Acetate **↑** SCFAs**↓**	[51, 52, 53]
	*Helicobacteraceae*	LAB, *Muribaculaceae*	SCFAs **↓**	[54, 55]
	*Bacteroides*	‐	BAs (TCA, TDCA, TUDCA, TCDCA, T‐MCA), LCA, HCA**↑** SCFAs **↓**	[52, 54]
	‐	*Prevotella*	Guanosine triphosphate, LCA, HCA**↑** Creatine, l‐Carnitine **↓**	[50, 52]
	*Klebsiella*	*Lactobacillus*	LCA, HCA**↑**	[52, 54] [38, 56]
	*Allobaculum, Ruminococcaceae*	‐	Creatine, l‐Carnitine **↑** LCA, HCA**↓**	[52, 54, 56]
	*Clostridia, Lachnospiraceae*	‐	Uridine, Sphingosine‐1‐phosphate, S‐Acetyldihydrolipoamide‐E, Estradiol, Dopamine **↓**	[51, 54]
	*Dubosiella*, *Anaerostipes*	*Romboutsia*	LysoPC, 5‐HT **↑** PC **↓**	[56]
	*Blautia*	‐		[56]
	*Bacteroides*	*Clostridiales*, *Lactobacillales*, LAB	BAs**↑**	[52, 54]
AD patients	*Lactobacillus*	‐	GABA, LCA, AAs**↑**	[57, 58, 59]
	*Bifidobacterium*	‐	Lactic acid, GABA, Acetate **↑**	[57, 58, 60, 61]
	*Enterococcaceae*	*Roseburia, Gemmiger*, *Coprococcus*, *Butyricicoccus*	Butyric acid **↓**	[59, 61]
		*Faecalibacterium*	Butyric acid **↓**	[60, 61]
	*Blautia*	‐	SCFAs**↑**	[57, 58, 60]
		*Clostridium*, *Lanchnospiraceae*	SCFAs**↓**	[57, 59, 60, 61]
	*Akkermansia*	‐	SCFAs, Propionic acid **↑**	[57, 61]
	*Bacteroides vulgatus, Campylobacter jejuni*	‐	Glutamate metabolite 2‐oxoglutarate **↓**	[62]
	*Corynebacterium glutamicum, Brevibacterium lactofermentum, Brevibacterium avium*	‐	d‐Glutamic acid **↑**	[62]
	*Prevotella*	*Bacteroides* genera	Ammonia, Phenol, and p‐Cresol **↑** Isovaleric acid, Isobutyric acid, Formic acid **↓**	[58, 63]
	*Phascolarctobacterium*, *Gemella*, *Bacteroides*, *Alistipes*	*Bifidobacterium*, *Adlercreutzia*	‐	[60]
	*Ruminococcaceae*	*Bacteroidaceae, Veillonellaceae*	‐	[59]
	*Escherichia, Streptococcus, and Enterococcus*	*Alistipes*	‐	[58]
AD mice	*Bacteroides intestinalis*, *Bacteroides fragilis*, *Bacteroides xylanivsolvens*	‐	AA, PE, 1‐St‐2‐Ln‐GPE, Arginine metabolite **↑**	[35]
	*norank_f_Muribaculaceae*, *Parasutterella, Agathobacter*	*Muribaculaceae*, *Atopobiaceae, Prevotellaceae*	Indole **↓**	[64]
	*Dubosiella*	‐	Cortical palmitoleic acid **↑**	[65]
	‐	*Enterobacter*	Acetate, BAs (DCA, IsoDCA, HDCA), LPS**↑** GABA, Propionate **↓**	[57, 65]
	*Akkermansia*	‐	UFA (11Z, 14Z, 17Z‐EA), Acetate**↑**	[65, 66]
	*Lachnospiraceae*	‐	Propionate **↑**	[57, 67]
		*Lactobacillaceae*	GABA, LCA, AAs**↓**	[57, 67]
	*Prevotella, Bacteroides*	*unclassified_f__Lachnospiraceae*, *Clostridium*	Pyruvate **↑**	[68]
	‐	*Blautia*	SCFAs**↓**	[57, 68]
	*Desulfovibrio*	*Bacteroides S24‐7 group*, *Alloprevotella*	Butyrate **↓**	[66]
	*Desulfovibrio* C21_c20	*Butyricicoccus pullicaecorum*	SCFAs**↓**	[69]

Abbreviations: AA, arachidonic acid; AAs, amino acids; *A. muciniphila*, *Akkermansia muciniphila*; BAs, bile acids; BCAA, branched‐chain amino acids; BHB, β‐hydroxybutyrate; DCA, deoxycholic acid; HCA, hydroxybutyric acid; HDCA, hyodeoxycholic acid; IsoDCA, 3β‐hydroxydeoxycholic acid; LAB, lactic acid bacteria; LCA, lactic acid; LysoPC, lysophosphatidylcholine; PC, phosphatidylcholine; PE, phosphatidylethanolamine; TCA, taurocholic acid; TCDCA, taurochenodeoxycholic acid, TDCA, taurodeoxycholic acid, T‐MCA, T‐methylcholanthrene acid; TUDCA, tauroursodeoxycholic acid; UFA, unsaturated fatty acid; 1‐St‐2‐Ln‐GPE, 1‐stearoyl‐2‐linoleoyl‐glycerophosphoethanolamine; 17Z‐EA, 17Z‐eicosatrienoic acid.

The gut microbiota, a complex and delicate microbial ecosystem within the human body, is crucial for maintaining host health, with its stability and diversity playing vital roles [72]. Studies have shown that significant changes occur in the gut microbiota composition of patients with both T2DM and AD, characterized by a trend of decreased probiotics and increased pathogens [37]. Dysbiosis of the gut microbiota exerts profound impacts on the onset and progression of T2DM and AD, not only by modulating metabolic pathways, immune function, and inflammatory responses, but also through the mediation of the gut‐brain axis [73].

The gut‐brain axis, as a critical pathway connecting the gut and the brain, plays a pivotal role in regulating the pathological processes of diabetes, inflammation, and AD via gut microbiota‐derived metabolites, including short‐chain fatty acids (SCFAs) [74]. In T2DM, gut microbiota dysbiosis disrupts insulin signaling and neurotransmitter balance, thereby affecting glucose homeostasis [75]. In AD, it potentially facilitates the secretion of inflammatory mediators and exacerbates neuronal injury, thereby accelerating the progression of cognitive decline [76]. Therefore, the regulatory role of the gut‐brain axis offers a novel perspective for the combined treatment of T2DM and AD. Below, we will separately discuss the common effects of various phyla of gut bacteria and their metabolites on both T2DM and AD.

#### The *Bacteroidetes* phylum and its metabolites

Alterations in the microbial composition within the *Bacteroidetes* phylum are closely associated with metabolic disorders and inflammatory reactions in both T2DM and AD. In animal models of T2DM, elevated levels of *Prevotella copri* (*P. copri*) and *Bacteroides vulgatus* (*B.vulgatus*) contribute to the exacerbation of IR via enhanced biosynthesis of branched‐chain amino acids (BCAAs) [49]. A cross‐regional metagenomic analysis involving 8117 samples from diverse cohorts confirmed the pathogenic potential of this relationship. Mei et al. identified strain‐specific gut microbial signatures in T2DM, emphasizing the persistent enrichment of *Prevotella* spp. associated with BCAAs metabolism across different populations [77]. Studies have shown that elevated BCAAs levels or alterations in their metabolic profiles are closely linked to T2DM and can significantly predict its onset [78, 79, 80]. Notably, the BCAAs biosynthesis capacity of *Prevotella* spp. depends on specific subpopulations and populations, which may be difficult to capture through traditional community‐level classification or microbial functional analysis [77]. Similarly, in AD, *B. vulgatus* in the gut promotes Aβ deposition and neuroinflammation through the production of lipopolysaccharide (LPS), while a decrease in *Prevotella* spp. abundance increases intestinal permeability, exacerbating LPS entry into the bloodstream and inducing synaptic damage and cognitive decline [81, 82, 83, 84]. Related research also suggests that LPS in the blood of AD mice can cause synaptic loss, neuronal death, and memory impairments, and may induce cognitive dysfunction in humans [85]. Furthermore, LPS may also participate in the pathophysiological processes of AD by promoting Aβ pathology, p‐Tau pathology, and microglial activation [86]. From this, it is evident that specific strains within the *Bacteroidetes* phylum, such as *P. copri* and *B. vulgatus*, drive metabolic disorders and neuroinflammation in T2DM and AD, respectively, through a bidirectional mechanism involving their metabolites (BCAAs, LPS). This reveals the cross‐disease regulatory feature of gut microbiota known as “one bacterium, multiple effects.”

#### The *Firmicutes* phylum and its metabolites

Firstly, the study by Wu et al. demonstrated notable changes in the prevalence of potential butyrate‐producing bacteria in patients with prediabetes and T2DM, underscoring the essential contribution of gut microbiota to the development of metabolic disorders [40]. Butyrate, a crucial metabolic product, exhibits notable effects in promoting insulin secretion and reducing blood glucose concentrations [87]. However, it is noteworthy that in T2DM patients, there is a decreasing trend observed in the abundance of the majority of butyrate‐producing bacteria, including species such as *Faecalibacterium*, *Clostridium*, *Oscillibacter*, and *Roseburia*, exhibits a declining trend. These bacteria play central roles in the processing of DNA, RNA, and proteins, and their overexpression may adversely affect insulin sensitivity, exacerbate inflammatory responses, and disrupt metabolic pathways, thereby posing a threat to insulin sensitivity [40]. On the other hand, a study involving 2166 participants revealed a strong correlation between elevated microbial α‐diversity and the abundance of butyrate‐producing bacteria (e.g., *Christensenellaceae*, *Ruminococcaceae*, *Clostridiaceae*, and *Peptostreptococcaceae*), and a reduced risk of T2DM as well as alleviated IR [57]. This finding is consonant with the research results of Ruuskanen et al. [88]. Similarly, studies on AD have reported a notable reduction in the abundance of *Firmicutes* and beneficial bacterial species, including butyrate‐producing bacteria, within the gut microbiota of AD patients [60, 89, 90]. Butyrate possesses the potential to reverse neurofibrillary damage and cognitive impairments [91]. These changes in microbial community structure not only compromise the integrity of the intestinal epithelial barrier, but they may also elicit the translocation of neuroinflammatory responses, thereby accelerating the deposition of Aβ plaques and subsequently exacerbating the pathological progression of AD [35, 92]. Therefore, the decreased abundance of butyrate‐producing bacteria (such as *Faecalibacterium* and *Roseburia*) not only serves as a common pathological marker for insulin resistance in T2DM and Aβ deposition in AD, but also directly links the metabolic and neurodegenerative pathways in these two diseases through the dual roles of their metabolite, butyrate, in maintaining intestinal barrier integrity and inhibiting systemic inflammation. The reduction in α‐diversity of the gut microbiota further amplifies this risk [57].

Furthermore, the genus *Lactobacillus* exhibits a crucial protective role in both T2DM and AD. In T2DM, diverse strains of *Lactobacillus*, including *Lactobacillus plantarum*, *Lactobacillus paracasei*, and *Lactobacillus fermentum*, exert beneficial effects on the gut microbiota composition by elevating the proportion of *Bacteroidetes* and reducing that of *Firmicutes*. These strains also stimulate the synthesis of SCFAs, which not only restore the integrity of the intestinal mucosal barrier but also enhance hepatic antioxidant enzyme activity, thereby safeguarding against oxidative damage. Furthermore, by modulating glucose metabolism pathways in the liver, these *Lactobacillus* strains effectively preserve the structure and function of pancreatic β‐cells, significantly attenuating the pathological progression of T2DM [93, 94, 95, 96, 97, 98, 99, 100, 101]. Similarly, studies have shown that the reduction of amino acid‐metabolizing genera (such as *Lactobacillus* and *Clostridium*) exacerbates the pathological characteristics of AD in mouse models [58, 64, 102, 103]. The research by Pan et al. further confirms that diets rich in probiotics such as *Lactobacillus* can improve cognitive decline, reduce Aβ load, and inhibit excessive glial cell activation by regulating metabolites, lowering carbohydrate metabolism, and increasing the abundance of amino acids such as sarcosine and dimethylglycine, thereby exerting a protective effect on AD [104]. These results, which demonstrate strain‐specific functions, highlight the importance of targeting functional subgroups.

Notably, the tryptophan metabolite indole‐3‐propionic acid (IPA) exhibits protective effects in both T2DM and AD. In T2DM, elevated levels of IPA mitigate the risk of diabetes through the inhibition of pro‐inflammatory gamma‐linolenic acid (GLA) pathways [105, 106, 107, 108, 109, 110]; whereas in AD, the lack of IPA exacerbates oxidative stress and synaptic damage [111]. Therefore, the modulation of gut microbiota composition, specifically by augmenting the population of potential butyrate‐producing bacteria and *Lactobacillus* species, along with the elevation of beneficial metabolites like IPA, may offer novel avenues and approaches for the prevention and management of T2DM and AD.

#### Other bacterial phyla and metabolites

Within the *Actinobacteria* phylum, the genus *Bifidobacterium* has demonstrated significant impacts on disease progression in both T2DM and AD, regulating the balance of the gut microbiota and inflammatory pathways. In T2DM, despite a general reduction in gut microbiota diversity, an enhanced abundance of *Bifidobacterium* is associated with increased serum levels of LPS and the anti‐inflammatory cytokine IL‐10, suggesting a potential indirect improvement in IR by antagonizing the pro‐inflammatory effects of LPS [112, 113]. Furthermore, a study by Gurung et al. showed a negative correlation between *Bifidobacterium* and T2DM [114]. A meta‐analysis by Koutnikova et al., including 105 randomized controlled trials (RCTs) with intervention durations exceeding 14 days and involving 6826 participants, robustly confirmed the significant positive effects of *Bifidobacterium* on reducing fasting blood glucose, glycated hemoglobin (HbA1c), insulin levels, and improving IR in T2DM patients [115]. Additionally, supplementation with *Bifidobacterium* breve (*B. breve*) significantly decreased HbA1c, low‐density lipoprotein (LDL), triglyceride (TG) levels, as well as blood urea nitrogen and creatinine concentrations in T2DM patients, while altering their gut microbiota composition, which is beneficial for T2DM management [116]. Similarly, in AD, a reduction in the abundance of *Bifidobacterium* is linked to heightened neuroinflammation and cognitive deterioration, while administration of *Bifidobacterium* species, including *B. breve* and *Bifidobacterium longum*, can alleviate cognitive deficits by suppressing gut inflammation, decreasing Aβ deposition, and improving blood‐brain barrier integrity [117, 118, 119, 120, 121, 122, 123, 124]. Therefore, supplementing with *B. breve*may offer protective effects against both T2DM and AD.

Moreover, other genera within the *Actinobacteria* phylum also exhibit protective effects on T2DM and AD. For instance, *Bifidobacterium animalis* significantly improves glucose homeostasis and glucose tolerance in T2DM rats, reduces structural damage to the pancreas and liver, decreases hepatic fat deposition, alleviates inflammation and oxidative stress levels, and regulates hepatic glucose metabolic pathways [125]. Specific strains such as *Bifidobacterium longum* BB68S and *Bifidobacterium lactis* Probio‐M8 positively impact AD pathology by modulating gut microbiota balance, reducing cerebral Aβ burden, ameliorating gut microbiota dysbiosis, and mitigating cognitive impairments [126, 127, 128, 129]. In summary, various *Bifidobacterium* species hold potential therapeutic value for both T2DM and AD.

Within the *Verrucomicrobia* phylum, *Akkermansia muciniphila* (*A. muciniphila*) can concurrently reduce the inflammatory state in both T2DM and AD [130, 131]. However, a reduction in the abundance of *A. muciniphila* is observed in the gut microbiota of individuals afflicted with these two diseases, a phenomenon that is intimately associated with impaired insulin secretion, disturbances in glucose homeostasis, and disruptions to bile acid metabolism [132]. As a quintessential probiotic species, *A. muciniphila* demonstrates a significant efficacy in reducing fasting blood glucose levels among patients with T2DM, enhancing glucose tolerance, and contributing to the prevention and management of T2DM via multifaceted mechanisms. These include the improvement of metabolic processes, mitigation of inflammation, enhancement of gut barrier integrity, and maintenance of microbial homeostasis, ultimately leading to a reduction in disease risk [133, 134]. In AD, *A. muciniphila* may be associated with enhanced cerebrovascular function and reduced AD risk [135]. Specifically, a reduction in *A. muciniphila* numbers alters bile acid composition, characterized by decreased levels of primary bile acids (e.g., cholic acid) and increased levels of secondary bile acids (e.g., deoxycholic acid) produced by gut microbiota metabolism. These changes in bile acid composition correlate with cognitive decline in AD and may exacerbate neuroinflammation and Aβ deposition, adversely affecting AD progression [136, 137, 138]. Additionally, research by Nishiwaki et al. indicates that *A. muciniphila* exerts neuroprotective effects on the brain by secreting SCFAs and reducing neuroinflammation [131]. Notably, the gut microbiota may undergo dynamic changes as AD progresses, which could further influence the efficacy of *A. muciniphila* Furthermore, various environmental factors, including dietary habits, lifestyle patterns, medication regimen, and individual genetic predispositions, can exert modulatory effects on the influence of *A. muciniphila* and its metabolic products on the pathology of AD, thereby rendering its contribution to AD a more intricate and multifaceted phenomenon [139]. Therefore, when exploring the relationship between *A. muciniphila* and T2DM or AD, it is crucial to consider multiple factors comprehensively to fully elucidate its potential biological effects.

Within the *Proteobacteria* phylum, *Escherichia coli* (*E. coli*) exhibits significant pro‐inflammatory and metabolic regulatory roles in the pathological processes of both T2DM and AD. In T2DM, *E. coli* significantly enriches in the gut during the prediabetic stage, and its increased abundance correlates with the accumulation of antibiotic resistance genes (such as multidrug resistance genes), directly exacerbating metabolic complications such as IR and atherosclerosis, while serving as a biomarker for predicting and diagnosing T2DM and its prediabetic stage [77, 140, 141]. In AD, an excessive proliferation of *E. coli* species stimulates the production of Aβ and the release of endotoxin LPS. This, in turn, results in heightened levels of pro‐inflammatory cytokines, including interleukin‐1β (IL‐1β) and tumor necrosis factor α (TNF‐α), in both the blood and brain. Consequently, this triggers neuroinflammation, promotes Aβ deposition, and exacerbates tau protein pathology, ultimately accelerating the progression of cognitive decline [58, 89, 142, 143]. Furthermore, the enrichment of *E. coli*‐derived LPS in the neocortex and hippocampus further disrupts blood‐brain barrier function, exacerbating synaptic damage and neuronal death, highlighting its common mechanism of driving pathological progression through the inflammation‐metabolism axis in both diseases [90, 144]. Therefore, *E. coli* within the *Proteobacteria* phylum exacerbates insulin resistance in the prediabetic stage of T2DM by harboring antibiotic resistance genes, and promotes Aβ deposition and neuroinflammation in AD by releasing LPS. The disruption of the inflammation‐metabolism axis driven by *E. coli* may be one of the core factors underlying the comorbidity of these two diseases.

### The differential roles of gut microbiota and metabolites on T2DM and AD

#### The bacteroidetes phylum and its metabolites

The pathogenic roles of the *Bacteroidetes* phylum in T2DM and AD exhibit differences in metabolic disturbances. For instance, in a gut microenvironment dominated by *Bacteroidetes*, the histidine (His) metabolism pathway in T2DM patients undergoes significant alterations, leading to abnormally elevated levels of imidazole propionic acid (ImP) in the body, which further impairs glucose metabolism [145]. Studies have shown that ImP positively correlates with glucose metabolism disorders and systemic inflammation, directly contributing to IR [145, 146]. His, an indispensable amino acid in the diet, cannot be endogenously synthesized by the body and thus must be acquired through dietary intake [147]. It is noteworthy that this adverse effect is not directly caused by dietary His but rather is a consequence of abnormal intestinal microbial metabolism. Conversely, research by Warmburnn et al. revealed that the administration of exogenous His supplementation exerts a beneficial impact on glycemic control in patients with T2DM [148]. Specifically, it led to a reduction in fasting blood glucose levels, HbA1c levels, and the extent of glycemic fluctuations, while enhancing insulin sensitivity. This further confirms the complex interplay between intestinal microbial metabolism and T2DM. Moreover, *Bacteroides ovatus* (*B.ovatus*) disrupts vitamin B12 (VB12) absorption, exacerbating VB12 deficiency and increasing the risk of cardiovascular disease, while *Alistipes* spp. and *Parabacteroides distasonis* exert protective effects by improving intestinal barrier function or regulating inflammatory responses [149, 150, 151]. Additionally, fecal microbiota transplantation (FMT) experiments in T2DM patients have shown that even with reduced abundances of *Bacteroidaceae*, the overall microbial community retains the pathogenic potential to induce hyperglycemia [50]. These results highlight the complexity of microbial functions and the specificity of their interactions with host metabolism.

In AD, further research by Xia et al. confirmed that the transplantation of the fecal microbiota from AD patients into mice resulted in a marked elevation in the abundance of the pro‐inflammatory species *B. vulgatus* [152]. This increase was tightly linked to the production of (5E,8E,10E)‐12‐hydroxyheptadecatrienoic acid (12‐HHTrE) and prostaglandin E2 (PGE2), both metabolites of polyunsaturated fatty acids (PUFAs). Notably, while PUFAs typically convert into prostaglandins and leukotrienes with anti‐inflammatory effects through cyclooxygenases (COX) and lipoxygenases (LOX) in the body, exerting positive effects on AD [153], Chen et al. reported that following the transfer of the gut microbiota from AD patients into germ‐free mice, the number of *Bacteroidetes* phylum associated with PUFA metabolism increased in the mice, which was related to microglial activation and inflammatory processes [35]. This finding implies that, although PUFAs play a role, alterations in the composition of the gut microbiota are not exclusively attributable to PUFAs. Rather, they may also be linked to reductions in the abundance of other genera, including *Prevotella* and *Barnesiella*, which collectively contribute to heightened intestinal inflammation and elevated levels of inflammatory cytokines in both the colon and plasma [154]. This suggests the consequences of a synergistic imbalance among multiple bacterial species.

Additional study has shown that the proportion of *Bacteroides* progressively rises over time in mouse models of AD [155]. As major producers of γ‐aminobutyric acid (GABA), *Bacteroides* not only maintain intestinal integrity but may also participate in inhibiting vagal nerve signaling, thereby potentially exerting positive effects on AD [156]. However, research by Zhao et al. found that the abundance of *Bacteroides* in the gut microbiota of AD mouse models actually decreased and was closely associated with Aβ burden [157]. The administration of exogenous *B. ovatus* or its metabolite, lysophosphatidylcholine, led to a significant decrease in Aβ burden and an enhancement of cognitive function, thereby underscoring the potential merit of targeted, strain‐specific interventions. In contrast, the role of *B. ovatus* in VB12 metabolism regulation in T2DM has not been clearly reported in AD.

#### The *Firmicutes* phylum and its metabolites

Firstly, the impact of the *Lachnospiraceae* family within the *Firmicutes* phylum exhibits differential effects on T2DM and AD. Studies have shown that in T2DM, unclassified *Lachnospiraceae* bacteria can reduce HbA1c levels and the risk of T2DM by lowering ketone body levels [158]. However, in AD, multiple genetic studies provide compelling evidence linking specific genetic signatures within the *Lachnospiraceae* family to an increased risk of AD [159, 160, 161]. Specifically, a significant correlation exists between decreased abundances of *Lachnoclostridium* spp. and *Roseburia hominis* and an elevated positivity rate of p‐Tau protein [161]. This reveals that the same bacterial family may exert bidirectional effects through differential regulation of metabolites in different diseases.

Furthermore, other genera within the *Firmicutes* phylum exert distinct effects on T2DM and AD, respectively. In T2DM, other strains within the *Lactobacillus* genus, such as *Lactobacillus rhamnosus*, *Lactobacillus reuteri*, *Lactobacillus gasseri*, *Lactobacillus brevis*, *Lactobacillus acidophilus*, and *Lactobacillus parabuchneri*, can alleviate insulin resistance, enhance insulin sensitivity, regulate blood glucose levels, improve epithelial barrier function, and modulate fasting and postprandial glucose levels [95, 162, 163, 164, 165, 166]. Additionally, a reduction in the abundance of the *Enterococcus* genus is accompanied by a reduction in serum melatonin concentrations, and this diminished melatonin secretion is independently linked to an elevated risk of T2DM [167]. Furthermore, the *Enterococcus* genus exhibits a negative correlation with serum biomarkers pertinent to T2DM, including IL‐1β, interleukin‐10 (IL‐10), TNF‐α, and LPS, as reported in the literature [112]. In contrast, high abundances of *Faecalibacterium*, *Butyricicoccus*, and *Dorea* in AD result in significant elevations of isobutyric acid levels in feces, serum, and the brain. These changes positively correlate with Aβ deposition and pro‐inflammatory cytokine levels, accompanied by cognitive decline [58, 61, 168]. By contrast, certain members of the *Clostridiaceae* family are associated with a reduced risk of AD [159], with high abundances of *Clostridium*, *Hungatella*, and *Eubacterium nodatum* being linked to higher cognitive function, while low abundances of *Megamonas* are associated with a shorter AD duration [58, 157, 160]. Furthermore, an elevation in the prevalence of other genera in *Firmicutes* phylum, notably *Clostridia*_UCG‐014, is accompanied by decreases in the abundances of *Faecalibacterium prausnitzii*, *Eubacterium rectale*, *Moryella*, and *Blautia*. These alterations are concomitant with heightened inflammatory responses, characterized by the upregulation of numerous inflammatory cytokines and a corresponding downregulation of the anti‐inflammatory cytokine IL‐10. This series of physiological alterations further correlates with increased Aβ deposition in the brain and elevated concentrations of phosphorylated p‐tau and Neurofilament Light Chain Protein (NfL) in plasma [142, 169, 170]. These findings further elucidate the complex impacts of the gut microbiota on T2DM and AD.

**FIGURE 1:**
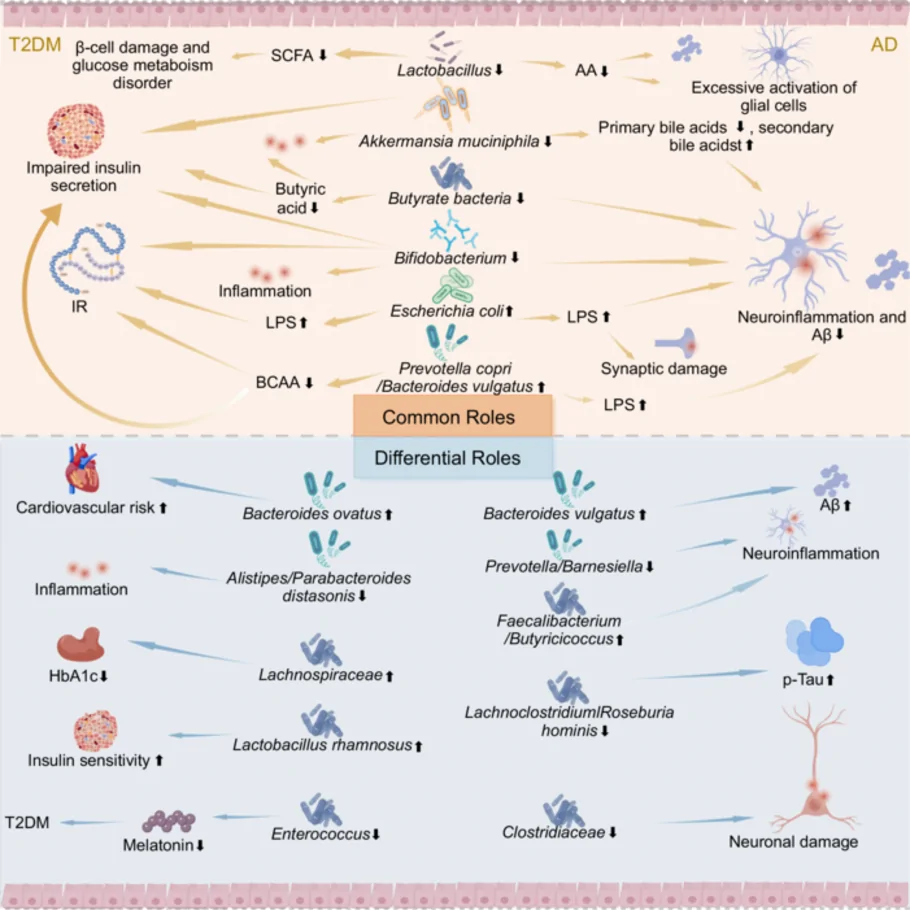
The common and differential roles of gut microbiota and their metabolites on type 2 diabetes mellitus (T2DM) and Alzheimer's disease (AD). AA, amino acid; Aβ, amyloid‐beta; BCAA, branched‐chain amino acid; HbA1c, glycated hemoglobin; IR, insulin resistance; LPS, lipopolysaccharide; p‐Tau, phosphorylated Tau; SCFAs, short‐chain fatty acids.

In summary, the communal and differential influence of gut microbiota and their metabolites on T2DM and AD is primarily manifested through their ability to modulate inflammatory responses, metabolic processes (such as the metabolism of BAs, BCAAs, and SCFAs), as well as the synthesis and regulation of neurotransmitters, thereby participating in and influencing the onset and progression of these two diseases. An alteration in the gut microbiota composition may contribute to the development of insulin resistance and persistent inflammation in T2DM, and concurrently, it may promote the onset and progression of AD through mechanisms involving enhanced blood‐brain barrier permeability and disrupted neuronal function. Figure 1 is a visualization of the above content.

## 3 MECHANISMS OF GUT MICROBIOTA AND METABOLITES IN THE PATHOGENESIS OF T2DM AND AD

Currently, studies examining the contribution of gut microbiota and their metabolic products to the etiology of T2DM and AD have largely centered on each disease in isolation, with a notable scarcity in investigations into their shared mechanisms in comorbidity. Given this background, the objective of the current study is to initially provide a comprehensive overview of the mechanisms through which gut microbiota and their metabolites are implicated in the pathogenesis of T2DM and AD, respectively. Building on the individual mechanistic insights, our investigation will delve into the potential shared pathways or interactions there between, with the aim of exploring potential mechanistic avenues for the therapeutic management of comorbidity between T2DM and AD through the modulation of gut microbiota and their metabolites. This endeavor seeks to open up new perspectives for research and treatment in this field.

### The mechanisms of gut microbiota and metabolites on T2DM

**FIGURE 2:**
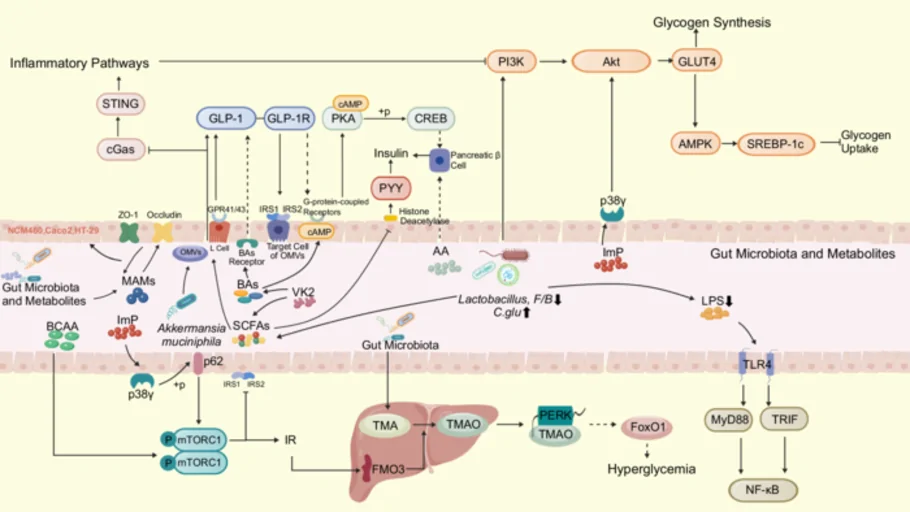
The mechanistic link between gut microbiota and its metabolites with type 2 diabetes mellitus (T2DM). Microbial anti‐inflammatory molecules (MAMs), originating from the gut microbiota and their metabolites, engage with proteins within the tight junction pathway, notably Zona Occludens‐1 (ZO‐1) and occludin, thereby bolstering the permeability and stability of intestinal epithelial cell lines, including NCM460, Caco2, and HT‐29. Bacteria such as *Akkermansia muciniphila*enhance glucose regulation by releasing outer membrane vesicles (OMVs) that elevate plasma glucagon‐like peptide‐1 (GLP‐1) levels, activate GLP‐1 receptors (GLP‐1R), and increase the expression of insulin receptor substrate 1 (IRS1) and IRS2 on OMV‐targeted cells. Additionally, they mitigate inflammatory responses by inhibiting the cyclic GMP‐AMP synthase (cGas) and stimulator of interferon genes (STING)‐mediated inflammatory pathway. An increased abundance of *Corynebacterium glutamicum* (*C. glu*), achieved by reducing the *Firmicutes*‐to‐*Bacteroidetes* (*F/B*) ratio and lactate‐producing bacteria, upregulates the IRS/phosphoinositide 3‐kinase (PI3K)/protein kinase B (Akt) signaling pathway, promoting the translocation of glucose transporter 4 (GLUT4) and glycogen synthesis, while further activating the AMP‐activated protein kinase (AMPK)/sterol regulatory element‐binding protein‐1c (SREBP‐1c) signaling pathway to inhibit glycogen uptake. Elevated short‐chain fatty acids (SCFAs) stimulate L‐cells to produce GLP‐1 by activating free fatty acid receptors G‐protein coupled receptor 41/43(GPR41/43) expressed on L‐cells, subsequently activating the GLP‐1R/cyclic AMP (cAMP)/protein kinase A (PKA)/cAMP response element‐binding protein (CREB) pathway to directly stimulate the release of insulin from pancreatic β‐cells. SCFAs also enhance the production of peptide tyrosine‐tyrosine (PYY) by inhibiting the activation of histone deacetylases. Arachidonic acid (AA) indirectly influences the secretion of insulin from pancreatic β‐cells through its metabolism. Bile acids (BAs) transduce the growth factor receptor 5 (TGR5)/cAMP/PKA/CREB signaling pathway to promote GLP‐1 secretion. Lipopolysaccharide (LPS) stimulates Toll‐like receptor 4 (TLR4)/myeloid differentiation factor 88(MyD88)/nuclear factor kappa‐light‐chain‐enhancer of activated B cells (NF‐κB) and TLR4/TIR domain‐containing adaptor inducing interferon‐β(TRIF)/NF‐κB signaling pathways, increasing inflammatory responses. Vitamin K2 (VK2) increases the levels of secondary bile acids, namely lithocholic acid and taurodeoxycholic acid, along with SCFAs such as acetic acid, butyric acid, and valeric acid. This enhancement significantly improves glucose tolerance through the activation of colonic bile acid receptors, modulation of the host's immune‐inflammatory responses, and an increase in circulating GLP‐1 levels. Branched‐chain amino acid (BCAA) overload may lead to insulin resistance (IR) by activating the mammalian target of rapamycin (mTOR), the catalytic subunit of mechanistic target of rapamycin complex 1 (mTORC1). IR induces the expression of flavin‐containing monooxygenase 3 (FMO3), facilitating the transformation of trimethylamine (TMA) into trimethylamine N‐oxide (TMAO). Elevated, pathogenic levels of TMAO have the capacity to directly interact with the endoplasmic reticulum stress kinase (PERK), leading to the activation of the transcription factor forkhead box protein O1 (FoxO1) and subsequently promoting hyperglycemia. Imidazole propionic acid (ImP) blocks the activation of AMPK by traditional antidiabetic drugs through the p38γ‐Akt pathway (where p38γ is a direct kinase of Akt). Furthermore, ImP promotes the phosphorylation of p62 via p38γ activation, subsequently activating the mTORC1 complex, interfering with the normal function of insulin receptors, and ultimately impairing insulin signaling.

The pathways through which gut microbiota and their metabolic products exert an influence on T2DM are illustrated in Figure 2.

#### Gut microbiota

Currently, most studies have confirmed that the efficacy of numerous drugs or plant extracts in managing T2DM is attributed to their ability to ameliorate hyperglycemia and IR by modulating the gut microbiota and associated alterations in metabolites, thereby alleviating T2DM [171, 172, 173]. Studies have shown that an increase in the abundance of gut microbiota, including *Lactobacillus* and *Ruminococcus* species, coupled with a decrease in the prevalence of *Desulfovibrio*, *Blautia*, *Parabacteroides*, and *Clostridium septicum*, can lead to a significant reduction in HbA1c, FBG, and IR levels in individuals with T2DM. Additionally, these changes are associated with the improvement of abnormal levels of pro‐inflammatory cytokines, such as TNF‐α and transforming growth factor‐β (TGF‐β) [174, 175, 176, 177, 178]. Mechanistically, this phenomenon is linked to microbial anti‐inflammatory molecules (MAMs) that originate from the metabolites of gut microbiota. These MAMs engage with proteins involved in the tight junction pathway, such as Zona Occludens‐1 (ZO‐1) and occludin. These interactions serve to enhance the permeability and stability of intestinal epithelial cell lines, including NCM460, Caco2, and HT‐29. Furthermore, they optimize the integrity of the intestinal barrier by upregulating ZO‐1 expression, thereby minimizing the leakage of harmful substances and mitigating systemic inflammatory responses [179, 180].

On the other hand, gut microbiota such as *A. muciniphila* exert profound effects on metabolic and inflammatory regulation through a unique mechanism involving the release of outer membrane vesicles (OMVs). The specific components within OMVs, such as Amuc‐1100, P9 (an 84 kDa protein secreted by *A. muciniphila*), and intercellular adhesion molecule‐2 (ICAM‐2), interact to not only elevate plasma levels of GLP‐1, facilitating cross‐talk between GLP‐1 receptor (GLP‐1R) and insulin signaling pathways, resulting in increased expression of insulin receptor substrate 1 (IRS1) and insulin receptor substrate 2 (IRS2) on target cells of OMVs and thus enhancing glucose regulation, but also to attenuate inflammatory responses by inhibiting the Cyclic GMP–AMP synthase (cGas) and stimulator of interferon genes (STING)‐mediated inflammatory pathways [181, 182]. Further, animal experimental results have demonstrated that the phosphoinositide 3‐kinase (PI3K)/protein kinase B (Akt) signaling pathway is closely associated with the cGas and STING‐mediated inflammatory processes [82, 183]. Inhibition of cGas‐STING activation can suppress the activation of the PI3K/Akt pathway [184, 185, 186]. Furthermore, alteration of the abundance of beneficial bacteria, such as *Lactobacillus* species, has been shown to decrease the mRNA expression levels of PI3K and Akt in the livers of mice, subsequently influencing hepatic and muscular glycogen metabolism in the context of T2DM [187]. However, research has demonstrated that by suppressing the *Firmicutes*‐to‐*Bacteroidetes* (*F/B*) ratio and markedly elevating the abundance of *Corynebacterium glutamicum* (*C. glu*), the IRS/PI3K/Akt signaling pathway becomes upregulated. This upregulation facilitates glycogen synthesis and enhances the translocation of glucose transporter 4 (GLUT4), ultimately leading to a significant improvement in glucose metabolic efficiency [188]. Research by Cui et al. also demonstrates that activating the IRS/PI3K/Akt signaling pathway can promote hepatic glycogen synthesis and glucose uptake, while activating the adenosine 5′‐monophosphate (AMP)‐activated protein kinase (AMPK)/Sterol regulatory element‐binding protein‐1c (SREBP‐1c) signaling pathway reduces hepatic lipid accumulation and inhibits glycogen uptake, exerting potential effects on T2DM‐induced glucose and lipid metabolic imbalances [189]. Similarly, at the metabolite level, both SCFAs and BCAAs have been shown to regulate the PI3K/AKT pathway and thereby exert beneficial effects on T2DM [190, 191]. Therefore, future research can prioritize examining the distinct impacts of PI3K/AKT pathways activated through inflammatory versus noninflammatory pathways on T2DM.

#### Short‐chain fatty acids

SCFAs are predominantly generated by members of the *Bacteroidetes* and *Firmicutes* phyla [192]. Changes in the composition of the gut microbiota, achieved by decreasing the *F/B* ratio and enriching the population of beneficial bacteria like *Parabacteroides*, *Bifidobacterium*, *A. muciniphila*, *Lactobacillus*, *Prevotella*, and various *Bacteroides* species, while simultaneously inhibiting harmful bacteria such as *Escherichia*/*Shigella*, certain *Lactobacillus* species, and *Desulfovibrio*, can lead to a significant elevation in the levels of SCFAs, including acetate, propionate, and butyrate. This, in turn, alleviates dyslipidemia, IR, reduces hepatic oxidative stress, and ameliorates jejunal damage—typical symptoms of T2DM [193, 194]. This mechanism may operate through the SCFAs‐GPR41/43‐GLP‐1 pathway [195]. Specifically, SCFAs have the capacity to stimulate L‐cells into producing GLP‐1 and peptide YY (PYY) by upregulating the expression of free fatty acid receptors GPR41 and GPR43 on the surface of L‐cells. This, in turn, enhances glucose homeostasis and promotes insulin secretion, thereby alleviating the symptoms of T2DM [196, 197, 198, 199, 200, 201]. This mechanism also enhances leptin release from adipocytes and influences hepatic vagal afferent nerve inhibition of feeding behavior, achieving weight management and improving IR [202]. Notably, beyond the GPR41 and GPR43 receptor pathways, SCFAs promote PYY production through other mechanisms, such as inhibiting the activation of histone deacetylases, although this effect may differ between humans and mice [203].

Similarly, Xia et al. utilized colonic transcriptome data and immunohistochemical findings to reveal that the pathway by which SCFAs promote GLP‐1 release also involves GLP‐1/GLP‐1R/cyclic adenosine monophosphate (cAMP)/protein kinase A (PKA)/cAMP response element‐binding protein (CREB) [204]. Upon binding to GLP‐1R, activated GLP‐1R increases intracellular cAMP levels through a G‐protein‐coupled receptor mechanism. The elevated cAMP activates PKA, which in turn phosphorylates and activates CREB. Phosphorylated CREB, functioning as a transcription factor, translocates to the nucleus where it modulates the transcription of relevant genes, including facilitating the expression and secretion of insulin‐encoding genes [205, 206]. Through the activation of the aforementioned signaling pathway, GLP‐1 exerts a direct effect on pancreatic β‐cells, stimulating the secretion of insulin and consequently elevating the levels of insulin in the circulation [207].

#### Unsaturated fatty acids

In T2DM, uniform federal accessibility standards (UFAs) primarily derive from dietary sources, whereas changes in the composition and functionality of the gut microbiota may impact their absorption and utilization. UFAs are involved in the regulation of blood glucose and lipid metabolism in individuals with T2DM through various pathways. Zhou et al. performed a comprehensive examination of the association between gut microbiota dysbiosis and T2DM, demonstrating that the restoration of gut microbiota equilibrium, specifically through the augmentation of beneficial bacteria like *Parasutterella* and the reduction of harmful bacteria such as *Alistipes*, *Odoribacter*, and *Anaeroplasma*, can effectively modulate the levels of UFAs, including α‐linolenic acid, linoleic acid, and arachidonic acid (AA), in fecal samples [208]. This alteration not only facilitates an elevation in high‐density lipoprotein cholesterol levels but also markedly enhances the lipid profile and glycemic management in mice with diabetes [95]. Among these, linoleic acid has been identified as a key UFA metabolite closely associated with hypoglycemic effects. Its presence enhances intestinal GLP‐1 release and insulin secretion within the pancreas, effectively alleviating IR and achieving glycemic control [209, 210].

Furthermore, AA serves as a pivotal metabolite in the interplay between probiotics‐induced alterations in lipid profiles, insulin sensitivity, and inflammation [211]. Hou et al. utilized metabolomics approaches to investigate how enhancing AA metabolism could represent a novel therapeutic target for glycemic control [194]. Studies have demonstrated that AA plays a pivotal role in modulating β‐cell function during the aging process, exerting positive effects on T2DM by promoting the production of lipoxin A4 (LXA4), inhibiting the generation of reactive oxygen species (ROS), and counteracting the cytotoxicity induced by streptozotocin (STZ) and alloxan, thus contributing to the preservation of β‐cell function [212, 213, 214, 215]. By targeting AA metabolism, future research may uncover innovative strategies to enhance β‐cell health and improve glycemic control in patients with diabetes.

#### Amino acids

Research indicates that BCAAs are linked to particular bacterial species, including *P. copri* and *B. vulgatus* [216]. The metabolic processing of BCAAs is markedly diminished in the adipose tissue of individuals with insulin resistance, resulting in the accumulation of excess fat within adipocytes and subsequently worsening insulin resistance. Additionally, the elevated production of BCAAs by the gut microbiota, coupled with the reduced expression of enzymes responsible for BCAAs metabolism in white adipose tissue, are implicated as contributors to the heightened serum BCAAs levels observed in insulin‐resistant conditions [217]. The findings by Zhang et al. reveal a notable positive association between the relative abundance of *Butyricicoccus* and the concentrations of BCAAs, as well as the Homeostasis Model Assessment of Insulin Resistance scores [218]. Notably, previous studies have shown that decreased BCAAs levels, particularly of l‐valine and l‐isoleucine, are associated with improved metabolic health [219]. Zheng et al. demonstrated that modulation of the altered abundance of bacterial genera associated with serum and fecal BCAAs, including *Blautia*, *Dubosiella*, *Lachnoclostridium*, *Lachnospiraceae*_NK4A136, *Oscillibacter*, and *Roseburia*, can effectively suppress BCAAs biosynthesis in the intestinal environment [220]. This adjustment of the microbial community not only enhances the catabolism of BCAAs but also upregulates the expression of tissue‐specific enzymes in vivo, leading to improved blood glucose and insulin levels and diminished expression of inflammatory cytokines. Moreover, the mechanistic target of rapamycin complex 1 (mTORC1) is activated by BCAAs, in conjunction with insulin and glucose, via increased cellular adenosine triphosphate (ATP) availability. BCAAs overload may lead to IR) by activating the catalytic subunit of mTORC1, namely mammalian target of rapamycin (mTOR), and resulting in increased acylcarnitines. In this context, mTOR is considered a central signal for crosstalk between BCAAs and insulin [221].

Moreover, the downregulation of diaminopimelic acid expression exerts a critical function in modulating dysfunctional glucose metabolism, particularly by influencing the expression of the key hepatic gene glycerol‐3‐phosphate acyltransferase 3 (Gpat3) [222]. Gpat3, as a crucial protein closely associated with gastrointestinal tract damage in T2DM, has its expression levels significantly affected by specific gut microbiota such as *Firmicutes*, *Bacteroidetes*, and *Clostridium* species [223, 224]. These findings imply the presence of an intricate interplay mechanism between the gut microbiota and the expression of Gpat3, ultimately affecting glucose metabolism and gastrointestinal wellbeing.

In addition, the hyperglycemic environment may act as an inhibitory factor, reducing the activity of tryptophanases and consequently affecting the microbial conversion pathways of tryptophan, such as by decreasing the production of skatole, a microbial derivative of tryptophan. The decrease in skatole compromises its capacity to stimulate the retinoic acid‐inducible gene‐I (RIG‐I)‐like receptor (RLR) signaling cascade. Within this pathway, laboratory of genetics and physiology 2, a pivotal component of the RLR family, initiates a sequence of downstream signal transmission events by interacting with mitochondrial antiviral signaling protein, which includes the activation of NF‐κB, ultimately leading to enhanced inflammation and exacerbation of the pathological progression of T2DM [225, 226, 227]. Therefore, improving AA metabolism can effectively ameliorate T2DM [194, 228].

#### Other lipid metabolites

As amphipathic molecules, BAs not only promote the digestion of dietary lipids but also serve as signaling molecules regulating lipid and glucose metabolism, as well as modulate the composition of the gut microbiota in the host organism [229]. Firstly, studies by Chen et al. revealed that alterations in the prevalence of intestinal anti‐inflammatory bacteria and opportunistic pathogens can influence the functional characteristics of the gut microbiota in rats with T2DM, ultimately leading to the upregulation of BAs biosynthesis [230]. In particular, genera such as *Prevotella*, *Alistipes*, and *Ruminococcus* are closely associated with BAs production. These BAs, encompassing chenodeoxycholic acid (CDCA), cholic acid (CA), and taurine‐conjugated bile acids (T‐BAs), modulate endoplasmic reticulum stress in the liver by suppressing the FXR/neuronal ceramide signaling axis and activating the TGR5/cAMP/PKA/CREB signaling cascade via the stimulation of residual FXR and TGR5 receptors across multiple organs. This activation enhances the secretion of GLP‐1, thereby inhibiting hepatic gluconeogenesis, preserving pancreatic β‐cells, regulating blood glucose concentrations, and effectively mitigating T2DM [42, 231, 232]. Furthermore, studies by He et al. and Tao et al. have demonstrated that particular bacterial species, including *Lactobacillus* and *Ruminococcus*, possess the capability to decrease the concentrations of detrimental deoxycholic acid (DCA) and lithocholic acid (LCA) [233, 234]. Additionally, *Streptococcus* and *Bacteroides* exert an influence on the balance between CA and ursodeoxycholic acid (UDCA), thereby facilitating the activation of the FXR/fibroblast growth factor 15 (FGF15) signaling pathway. This, in turn, enhances insulin sensitivity, thereby improving the pathological state of T2DM [233, 235].

Moreover, the findings of Tawulie et al. indicate that bacterial species possessing bile salt hydrolase activity, including *Bacteroides*, *Lactobacillus*, and *Bifidobacterium*, contribute to the accumulation of unconjugated BAs such as CDCA and DCA in the ileum, subsequently enhancing the activation of intestinal FXR/FGF15 and TGR5/GLP‐1 signaling pathways [236]. However, Zhu et al. found that in STZ‐induced T2DM rats injected with BAs, glucose tolerance was improved by activating the TGR5/GLP‐1 signaling pathway rather than the FXR/FGF15 pathway, thereby achieving glucose homeostasis [237]. This mechanism entails an elevation in the prevalence of *Firmicutes* and *Actinobacteria*, resulting in modifications to the fecal bile acid profile and an augmentation in the levels of UDCA, which subsequently stimulates and enhances TGR5 expression. Through the BA‐TGR5‐GLP‐1 pathway, this induces the expression of energy metabolism‐related genes in peripheral tissues, thereby increasing GLP‐1 secretion [238]. The research by Zhang et al. also supports this, revealing that FMT markedly enhanced glucose tolerance in mice with diet‐induced obesity through the activation of colonic bile acid receptors, the alleviation of host immune‐inflammatory responses, and the elevation of circulating GLP‐1 levels [239]. Furthermore, animal experiments have demonstrated that increasing the levels of glycohyocholic acid and DCA can also upregulate TGR5 expression and improve ileal epithelial damage. Meanwhile, TGR5 can regulate the abundance of L‐cells through Yes‐associated protein (YAP)‐driven intestinal regeneration in db/db mice to enhance GLP‐1 release, thereby maintaining glucose homeostasis and improving T2DM [240, 241].

Furthermore, BAs exert an influence on lipid metabolism and systemic inflammatory responses in individuals with T2DM via the gut‐liver axis. Changes in the gut microbiota composition and their metabolites, including an elevation in BA‐metabolizing bacteria such as *Lactobacillus* and *Bifidobacterium*, along with a reduction in SCFA‐producing bacteria like *Faecalibacterium*, *Bacteroides*, and *Roseburia*, may result in compromised intestinal barrier integrity, accompanied by alterations in protein expression patterns in the blood, intestine, and liver. These changes include upregulation of fibroblast growth factor receptor 4 (FGFR4) and transient receptor potential melastatin 5 (TRPM5), both of which are implicated in the homeostasis of BAs and lipid metabolism, along with the downregulation of Cytochrome P450, Family 27, Subfamily A, Polypeptide 1 (CYP27A1). Additionally, there are increases observed in Toll‐like receptor 6 (TLR6), myeloid differentiation factor 88 (MYD88), and NF‐κB, all of which are linked to inflammatory responses [242, 243].

LPS, a constituent of the Gram‐negative bacterial cell wall, plays a role in the initiation and advancement of T2DM by stimulating host inflammatory responses and promoting insulin resistance [33]. As the *F/B* ratio declines, concomitant with a decrease in the abundance of bacteria linked to aminoacyl‐tRNA biosynthesis, this shift is accompanied by an elevation in bacteria implicated in fatty acid and lipid biosynthesis, as well as sucrose metabolism. These alterations effectively diminish the concentrations of LPS and pivotal inflammatory cytokines, including interleukin‐6 (IL‐6), IL‐1β, and TNF‐α, in the bloodstream, liver, and adipose tissue. Consequently, this regulates the biosynthesis of LPS and its downstream signaling pathways, achieving a positive intervention in T2DM [244, 245]. The mechanism underlying these changes involves downregulation of mRNA expression levels of IkappaB kinase and NF‐κB within the hypothalamus, leading to a reduction in inducible nitric oxide synthase and inflammatory mediators, thereby regulating the inflammatory response and significantly promoting glycemic control [246, 247, 248].

Among the members of the TLR family, TLR4 possesses a unique dual signaling capability, recruiting two adapter proteins: MyD88 and the toll interleukin‐1 receptor‐domain‐containing adapter that induces interferon‐β (TRIF). MyD88‐mediated signaling triggers pro‐inflammatory innate immune responses, while TRIF‐mediated signaling leads to adaptive immune responses [249, 250]. Through the optimization of gut microbiota composition, the expression of LPS‐triggered TLR4/MyD88/NF‐κB and TLR4/TRIF/NF‐κB signaling cascades can be attenuated, ultimately leading to a decrease in cellular inflammatory responses and the retardation of T2DM progression [179, 251, 252, 253].

Alternatively, the refinement of the gut microbiota composition, involving the suppression of detrimental bacteria such as *Helicobacter pylori* and the promotion of probiotic proliferation, including species like *Allobaculum*, *Bifidobacterium*, and *Lactobacillus*, not only augments intestinal barrier function through the upregulation of mRNA expression of tight junction proteins, namely Claudin‐1, Occludin, and ZO‐1, but also attenuates the ectopic deposition of LPS, thereby impeding the progression of systemic inflammation and insulin resistance [254].

#### Vitamin

The gut microbiota, closely associated with dietary intake, such as *Lactobacillus* and *Bifidobacteria*, possess the capability for *de novo* synthesis and supply of vitamins [255]. Various categories of vitamins exert crucial roles in the development and progression of T2DM. Specifically, the production of vitamin B7 (Biotin) correlates with lower HbA1c levels and reduced baseline insulin requirements [256]. Various categories of vitamins play pivotal roles in the pathogenesis of T2DM. Specifically, the production of Biotin correlates with lower HbA1c levels and reduced baseline insulin requirements [257, 258]. Results from a Mendelian randomization study indicate that the effect of vitamin K (VK) levels on diabetes risk is not significant [259]. However, other studies reveal that VK2 not only enhances insulin sensitivity by participating in the synthesis of VK‐dependent protein osteocalcin, exerting anti‐inflammatory effects, and exhibiting lipid‐lowering actions, but also increases the concentrations of metabolites by elevating the abundance of bacterial genera that produce secondary bile acids (such as lithocholic acid and taurodeoxycholic acid) and SCFAs, including acetic acid, butyric acid, and valeric acid. This process significantly enhances glucose tolerance by activating colonic bile acid receptors, modulating the host's immunoinflammatory response, and elevating circulating concentrations of GLP‐1 [239, 260].

#### Trimethylamine N‐oxide

The gut microbiota, representing a intricate ecosystem resident within the, is capable of producing a variety of metabolites, among which trimethylamine (TMA) is an important precursor that undergoes metabolic conversion to Trimethylamine N‐oxide (TMAO) [261]. Recent research has revealed a strong correlation between heightened TMAO levels and the onset of T2DM, as well as its complications, such as chronic kidney disease (CKD) [262]. Specifically, patients with T2DM‐CKD display a marked elevation in the prevalence of gut bacteria that generate TMA, leading to excessive accumulation of TMAO and an elevation in intestinal permeability. These alterations may elevate the susceptibility to cardiovascular disease in patients with T2DM by intensifying chronic inflammation and impairing endothelial function [263]. Furthermore, TMAO not only acts as an indicator of T2DM risk but also actively contributes to the development of T2DM via multiple metabolic and immune pathways [264, 265]. It exhibits a strong correlation with elevated glucose and lipid levels, thereby shedding further light on the detrimental impact of TMAO in metabolic dysregulation [266, 267].

Upon chronic exposure to TMAO, T2DM mice exhibited exacerbated hepatic triglyceride accumulation and lipogenesis [268]. Elevated, pathogenic levels of TMAO have the capacity to directly interact with the endoplasmic reticulum stress kinase (PERK), triggering the activation of the PERK arm of the unfolded protein response (UPR), which subsequently leads to the activation of the transcription factor forkhead box protein O1 (FoxO1). This process is crucial for promoting the development of hyperglycemia [269, 270]. Of particular importance, IR, as a core feature of T2DM, can induce the expression of flavin‐containing monooxygenase 3 (FMO3), promoting the conversion of TMA to TMAO. Certain plant components, such as 3,3′‐diindolylmethane found in cruciferous vegetables, have been shown to effectively inhibit FMO3 activity, reduce TMAO levels, and decrease PERK activation, thereby exhibiting protective effects in insulin resistance models. This provides a new strategic perspective for the prevention and management of T2DM and its associated complications [271].

Other metabolites that have a mechanism of action on T2DM but not on AD.

A multicenter cohort analysis has revealed a significant elevation of serum ImP in patients with T2DM. ImP positively correlates with the abundance of symbiotic *Clostridium* and *Ruminococcus gnavus* species, as well as with glucose metabolism disturbances and systemic inflammation, directly contributing to IR [145, 146]. By activating the p38γ‐Akt pathway (where p38γ is a direct kinase of Akt), ImP can block the activation of AMPK by traditional antidiabetic drugs such as metformin. Notably, the administration of pirfenidone to specifically inhibit the activation of p38γ by ImP restores the antidiabetic effectiveness of metformin, thereby presenting a fresh strategic approach for the treatment of T2DM [272]. Furthermore, ImP promotes the phosphorylation of p62 through the activation of p38γ, a process that subsequently activates the mTORC1 complex, interfering with the normal function of insulin receptors and ultimately impairing insulin signaling, which exacerbates the pathological progression of T2DM [48]. More broadly, ImP also plays a detrimental role in wound healing processes in T2DM patients. It inhibits the spinster homolog 2 (SPNS2)‐mediated secretion of sphingosine‐1‐phosphate (S1P), thereby impeding the canonical activation of the Rho family of small GTPases (Rho) signaling cascade. This sequence of events markedly diminishes the angiogenic potential of human umbilical vein endothelial cells (HUVECs), consequently delaying the process of wound healing [273].

### The mechanisms of gut microbiota and metabolites on AD

**FIGURE 3:**
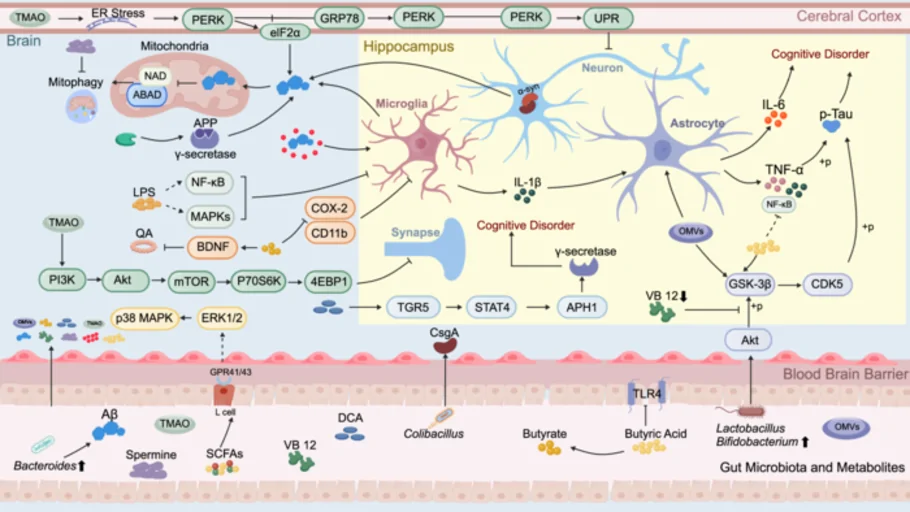
The mechanistic link between gut microbiota and its metabolites with alzheimer disease (AD). Increased abundance of *Bacteroides* exacerbates amyloid‐β (Aβ) deposition in the intestine, further promoting Aβ accumulation in the brain by inhibiting the phagocytosis of Aβ1‐42 injected into the hippocampus by microglia. Additionally, Aβ enters the mitochondrial matrix and accumulates, inhibiting amyloid β‐binding alcohol dehydrogenase (ABAD), thereby preventing its binding to nicotinamide adenine dinucleotide (NAD) and blocking mitophagy. The *curli fiber‐specific gene A* (*CsgA*) secreted by *Escherichia coli* colocalizes with α‐synuclein (α‐syn) within neurons, inhibiting Aβ deposition. An increase in *Lactobacillus* and *Bifidobacterium* populations also reduces Aβ deposition through phosphorylation regulation of the Akt/glycogen synthase kinase‐3β (GSK‐3β) pathway. Deficiency in vitamin B12 (VB12) disrupts the protein kinase B (Akt)/GSK‐3β signaling pathway within the hippocampus, indirectly elevating the concentrations of serum inflammatory markers tumor necrosis factor α (TNF‐α) and interleukin‐1 β (IL‐1β), thereby adversely impacting memory function in AD. NLRP3 proteins aggregate around Aβ in the intestine, activating microglia and leading to excessive IL‐1β production under systemic lipopolysaccharide (LPS) stimulation, which prompts astrocytes in the hippocampus to exhibit excessive chemotaxis to IL‐1β and produce interleukin‐6 (IL‐6), exacerbating cognitive impairment. Outer membrane vesicles (OMVs) induce tau phosphorylation by activating GSK‐3β and cyclin‐dependent kinase 5 (CDK5)‐calpain in the hippocampus. They also stimulate astrocytes and microglia, resulting in enhanced secretion of inflammatory cytokines, including NF‐κB, IL‐1β, and TNF‐α, within the hippocampus, ultimately culminating in cognitive impairment. Butyrate reduces the expression of TLR4 and NF‐κB, significantly alleviating the inflammatory response. Butyrate increases the expression level of brain‐derived neurotrophic factor (BDNF) in vivo, preventing the reduction of BDNF and related neuronal damage induced by quinolinic acid (QA). It also attenuates neuroinflammation through the reduction of integrin CR3 (CD11b) and cyclooxygenase‐2 (COX‐2) levels. Short‐chain fatty acids (SCFAs) stimulate GPR41 and GPR43 receptors on intestinal epithelial cells, which in turn activate the extracellular signal‐regulated kinase 1/2 (ERK1/2) and p38 mitogen‐activated protein kinase (p38 MAPK) signaling pathways, thereby exerting neuroprotective actions. Deoxycholic acid (DCA) induces upregulation of the bile acid receptor, Takeda G‐protein‐coupled receptor 5 (TGR5), which impairs cognitive function in AD by activating the phosphorylated Signal Transducer and Activator of Transcription 3 (p‐STAT3)/Anterior Pharynx Defective 1 Homolog 1 (APH1)/γ‐secretase signaling pathway. LPS‐induced phosphorylation of mitogen‐activated protein kinases (MAPKs) and NF‐κB stimulates neuroinflammation in BV2 microglia and the brain. Trimethylamine N‐oxide (TMAO) induces excessive endoplasmic reticulum (ER) stress in the cerebral cortex, resulting in elevated levels of pancreatic ER kinase (PERK) and the α‐subunit of eukaryotic translation initiation factor 2 (eIF2α). During ER stress, such as excessive accumulation of unfolded or misfolded proteins, binding immunoglobulin protein (BiP)/glucose‐regulated protein 78 (GRP78) dissociates from PERK, resulting in the activation of PERK through dimerization and autophosphorylation, initiating the unfolded protein response (UPR), which subsequently affects neuronal function. Furthermore, TMAO can cross the blood‐brain barrier, activate mTOR through the PI3K/Akt/mTOR signaling pathway, and induce decreased hippocampal synaptic plasticity through the mTOR/ribosomal protein S6 kinase 70 kDa (P70S6K)/eukaryotic translation initiation factor 4E binding protein 1 (4EBP1) pathway, subsequently affecting cognitive function.

The mechanisms by which the gut microbiota and their metabolites influence AD are depicted in Figure 3.

#### Gut microbiota

Mounting evidence indicates that intestinal dysbiosis may exert a substantial influence on the pathogenesis of AD, such as promoting Aβ aggregation [274], neuroinflammation [35], oxidative stress [275], and insulin resistance [276]. Studies have demonstrated that metabolic dysregulation in AD mouse models is linked to the gut microbiota, and modulating the gut microbial community by decreasing the prevalence of detrimental populations or elevating the levels of beneficial bacteria can improve cognitive function and learning capabilities in these animal models [82, 277]. Within the gut microbiota, an elevation in the prevalence of Bacteroides can augment the accumulation of Aβ in the intestine, subsequently facilitating its transport to the brain via the bloodstream, as opposed to the vagus nerve [278]. Additionally, *Bacteroides* can further aggravate Aβ deposition in the brain by inhibiting the phagocytosis of Aβ1‐42 injected into the hippocampus by microglia [279, 280]. Furthermore, Aβ enters the mitochondrial matrix and gradually accumulates within mitochondria, inhibiting mitochondrial enzymes such as amyloid‐β binding alcohol dehydrogenase (ABAD), thereby preventing its binding to nicotinamide adenine dinucleotide (NAD) and blocking mitophagy. This ultimately exacerbates neuronal damage, abnormal energy metabolism, and oxidative stress, accelerating disease progression and cognitive decline [281, 282, 283]. Conversely, *A. muciniphila* has the capacity to decrease the accumulation of Aβ1‐42 in the cerebral cortex and various brain regions [284]. The *curli‐specific genes A* (*CsgA*) secreted by *E. coli* colocalizes with α‐synuclein (α‐syn) within neurons and can promote α‐syn aggregation through cross‐seeding, significantly reducing the seeding activity of brain homogenates containing Aβ and inhibiting Aβ deposition [285, 286]. Moreover, increases in *Lactobacillus* and *Bifidobacterium* species can also restore AD‐related gut microbiota dysbiosis through phosphorylation regulation of the Akt/glycogen synthase kinase‐3β (GSK‐3β) pathway, reducing phosphor (Threonine 231)‐tau phosphorylation and Aβ deposition [287, 288, 289, 290, 291].

On the other hand, in addition to the endogenous gut microbiota, exogenous gut microbial communities can also exert an impact on AD. For example, following the transplantation of gut microbiota from AD patients into mice, an enhancement in the expression of nucleotide‐binding oligomerization domain‐, leucine‐rich repeat‐, and pyrin domain‐containing 3 (NLRP3) was noted in the intestinal tissue of the mice. These NLRP3 proteins aggregated around Aβ, activating microglia and, under systemic LPS stimulation, leading to excessive production of IL‐1β. This, in turn, prompted astrocytes to produce exaggerated chemotactic responses and IL‐6 in response to IL‐1β within the hippocampus, with upregulated expression of inflammatory cytokines exacerbating cognitive impairment [292, 293, 294]. Furthermore, the oral delivery of probiotics enhances glucose uptake and neuronal function in mouse models of AD by normalizing the expression of crucial glucose transporters (GLUT3, GLUT1) and insulin‐like growth factor receptor β in the brain, potentially through regulating the phosphorylation levels of signaling molecules such as AMPK and Akt [295].

Furthermore, OMVs are also linked to the onset of cognitive impairments, including learning and memory deficits, resulting from blood‐brain barrier (BBB) disruption in AD. OMVs not only trigger tau phosphorylation through the activation of GSK‐3β and cyclin‐dependent kinase 5 (CDK5)‐calpain in the hippocampus, but also stimulate astrocytes and microglia, thereby elevating the release of inflammatory cytokines (NF‐κB, IL‐1β, and TNF‐α) within the hippocampus, ultimately culminating in cognitive dysfunction [296, 297].

#### Short‐Chain fatty acids

Research has shown that the proportional abundance of bacteria capable of producing SCFAs, such as *Roseburia*, *Fusicatenibacter*, and *Erysipelotrichaceae_*UCG‐003, continues to decline in patients with AD [298]. A decrease in bacteria that produce SCFAs is linked to disruptions in the intestinal barrier, given the pivotal roles of SCFAs in intestinal motility, immune responses, and intestinal barrier function [299, 300, 301, 302]. Research has indicated that the expansion of butyrate‐producing bacteria, including those belonging to the *Enterobacteriaceae* family, can downregulate the expression of TLR4 and NF‐κB, significantly mitigating inflammatory responses [239]. This effect is attributed to butyrate's ability to attenuate LPS‐induced TLR4‐NF‐κB activation, blocking the NF‐κB signaling pathway by inhibiting the activation of TLR4 and RIG‐I receptors, and subsequently decreasing inflammation through the PI3K/AKT signaling pathway [290, 303]. Additionally, butyrate indirectly influences the phosphorylation level of Tau protein by acetylating the lysine 15 site of GSK‐3β, which regulates the phosphorylation status of its serine 9 site [117].

Butyrate derivatives, specifically butyrates, increase the expression level of BDNF in vivo through epigenetic regulatory mechanisms, specifically by enhancing histone H3 lysine 18 acetylation (H3K18ac) modification at the BDNF promoter. This, consequently, inhibits the decrease in BDNF and the ensuing neuronal damage induced by quinolinic acid (QA), which is generated from tryptophan via a cascade of enzymatic reactions [91]. Furthermore, butyrates exert their influence on the gut microbiota‐gut‐brain axis in AD by modulating the levels of integrin CR3 (CD11b) and cyclooxygenase‐2 (COX‐2), and by suppressing Aβ‐triggered phosphorylation of NF‐κB p65 in BV2 microglia, ultimately attenuating microglia‐associated neuroinflammation [304]. Among these, COX‐2 is a receptor associated with neuroinflammation, and inflammatory mediators, including IL‐1 and TNF‐α, can upregulate COX‐2 expression, thereby stimulating the production and release of prostaglandins and initiating a cascade of subsequent inflammatory reactions [305].

Furthermore, SCFAs derived from the gut microbiota are capable of activating GPR41 and GPR43 receptors on intestinal epithelial cells, which in turn trigger the activation of extracellular signal‐regulated kinase 1/2 (ERK1/2) and p38 mitogen‐activated protein kinase (p38 MAPK, also referred to as p38) signaling pathways within these cells. This activation process stimulates the production of chemokines and cytokines during immune responses, ultimately exerting neuroprotective effects in mouse models of AD [306, 307, 308].

#### Unsaturated fatty acids

Observational research has shown a negative association between the daily consumption of PUFAs and the likelihood of developing AD [309]. Research indicates that AD can be treated by modulating UFAs [310]. Furthermore, prostaglandin F2α (PGF2α), a prominent metabolite of AA, exhibits selective antagonistic effects on liver X receptors (LXRs)/retinoid X receptors α (RXRα) as well as RXR/RXR dimers. It effectively counteracts the clearance of Aβ by LXR agonist (TO901317) through inhibiting the expression of apolipoprotein E (APOE) and accelerates microglial inflammatory responses to Aβ or LPS [311]. TO901317 enhances cholesterol efflux through the activation of the LXR‐β/RXRα/ATP‐binding cassette transporter A1 (ABCA1) transmembrane transport system, leading to a reduction in caveolin‐1, APP, and β‐site amyloid precursor protein cleaving enzyme 1 (BACE1), ultimately resulting in decreased levels of Aβ42 in the brain [312].

#### Amino acids

Abnormal metabolites of AAs resulting from gut microbiota dysbiosis can mediate various causal effects of AD. Among them, decreased glutamine concentrations mediate the negative causal effect of *Holdenemania* species on AD, while increased alanine concentrations mediate the positive causal effect of *Parabacteroides* species on AD [313]. Elevated levels of 5‐HT, dopamine, and GABA can alleviate neuronal damage, Aβ deposition, and tau protein pathology [314]. Wang et al. reported that changes in the composition of the gut microbiota result in the peripheral accumulation of phenylalanine and isoleucine, which promotes the differentiation and expansion of pro‐inflammatory T helper 1 (Th1) cells [34]. These peripherally derived Th1 immune cells, upon infiltrating the brain, are linked to the activation of M1 microglia, thereby contributing to neuroinflammation associated with AD.

Furthermore, tryptophan metabolism serves as a potential source of endogenous anti‐AD molecules, capable of being chemically modified into multi‐target therapeutic modulators addressing the complex immunoproteinopathic mechanisms of AD [315]. The kynurenine pathway, a major catabolic route of tryptophan, results in the production of NAD and other neuroactive intermediates: QA and kynurenic acid (KA) [316]. Among them, QA exerts a range of toxic effects, encompassing overactivation of N‐methyl‐D‐aspartate (NMDA) receptors leading to excitotoxicity, disruption of synaptic function, and ultimately, neuronal death [317]. However, KA has been identified as the sole endogenous NMDA receptor antagonist, capable of modulating the neurotoxic effects of QA [317]. Additionally, tryptophan, through its metabolite indole‐3‐lactic acid (ILA), inhibits Aβ accumulation and cognitive impairment by activating microglial and astroglial cells and the aryl hydrocarbon receptor signaling pathway [318, 319].

#### Other lipid metabolites

BAs profile in AD may indicate early risks for AD progression [320]. Research indicates that the anabolic metabolism of BAs differs between individuals with AD and cognitively normal individuals, originating from the gut microbiota and subsequently transported to the brain [321]. BAs are primarily metabolized by *Clostridia* species, particularly *Clostridium scindens* [322, 323]. The study has demonstrated that in the initial stages of AD mouse models, DCA levels increase in the brain. Increased production of DCA triggers the upregulation of the bile acid receptor, known as Takeda G‐protein‐coupled receptor (TGR5), which disrupts cognitive function in AD by stimulating the phosphorylated signal transducer and activator of transcription 3 (p‐STAT3)/anterior pharynx defective 1 homolog 1 (APH1)/γ‐secretase signaling cascade [324].

LPS, a potent neurotoxic glycolipid in AD, can induce the phosphorylation states of p38, ERK1/2, c‐Jun N‐terminal kinase 1/2 (JNK1/2), and NF‐κB. The induced phosphorylation of mitogen‐activated protein kinases (MAPKs) and NF‐κB stimulates neuroinflammation in BV2 microglia and the brain [325, 326]. Furthermore, this mechanism may also involve activated p38 and JNK1/2, enhancing TNF‐α production in signal‐transducing Kupffer cells (KC) [327]. However, studies by Lloun et al. have demonstrated that LPS induces memory impairment and IR in the hippocampus of AD models, characterized by elevated levels of IRS1 and reduced Akt phosphorylation, but this effect is associated with p38, rather than JNK1/2 and ERK1/2 activation [328].

#### Vitamin

A tight correlation exists between vitamins and the progression of AD [329]. A study based on Mendelian randomization has revealed that specific gut microbiota, including *Lachnospiraceae* (NK4A136 group), *Defluviitaleaceae* (UCG011), and *Bifidobacterium*, serve as key mediators in the causal chain linking VB12, VB6, and B‐complex vitamins to the risk of AD [330]. Specifically, VB12 deficiency disrupts insulin signaling pathways within the hippocampus, particularly the Akt/GSK‐3β pathway, and alters the gut microbiota composition, further elevating the levels of serum inflammatory markers TNF‐α and IL‐1β, thereby adversely impacting memory function in AD [331]. However, another study [332] reportsthat high‐dose VB supplementation fails to effectively slow the decline in cognitive function in patients with mild to moderate AD. This finding may be attributed to the different roles played by endogenous and exogenous VB in the pathology of AD. Notably, VB12 serves as an essential cofactor for methionine synthase, playing a pivotal role in the regulation of the methionine/S‐adenosylmethionine (SAMe) cycle, a process that aids in mitigating the accumulation of Aβ [333]. Further research indicates that VB12 supplementation can reduce elevated homocysteine (Hcy) levels and alleviate the toxic effects of Aβ [334]. Importantly, oxidative stress triggered by increased Hcy levels is considered a key factor exacerbating Aβ toxicity. Therefore, vitamin B12, by influencing these complex biochemical pathways, demonstrates its potential value in modulating the pathological processes of AD.

#### Trimethylamine N‐oxide

TMAO is thought to mediate systemic inflammation and amyloidogenesis within the brain through endothelial dysfunction [335]. Endoplasmic reticulum (ER) stress is one of the important factors contributing to endothelial cell dysfunction. Studies have found that FMT from AD mice induces excessive ER stress in the cortex of recipient mice, manifested by increased levels of ER stress marker proteins such as PERK and the α‐subunit of eukaryotic initiation factor 2 (eIF2α) [336]. Similarly, Govindarajulu et al. applied an ex vivo model involving the incubation of wild‐type hippocampal slices with TMAO, and found that TMAO activated the PERK‐EIF2α‐ER stress signaling pathway in both the treated ex vivo slices and in db/db and 3×Tg‐AD mice [337]. Additionally, alterations in presynaptic receptor expression and decreases in postsynaptic receptor expression were noted. Consequently, TMAO is implicated in inducing synaptic plasticity impairments via the ER stress‐associated PERK signaling cascade, potentially contributing to the progression of AD [338]. Moreover, TMAO has the potential to elicit synaptic plasticity deficiencies through the PERK signaling pathway, which is mediated by ER stress [337]. When ER stress occurs, such as excessive accumulation of unfolded or misfolded proteins, binding immunoglobulin protein (BiP)/glucose‐regulated protein 78 (GRP78) dissociates from PERK, leading to the activation of PERK through dimerization and autophosphorylation. This activation triggers the UPR, ultimately influencing the expression of cyclin proteins in neurons implicated in AD [339, 340, 341].

Additionally, significantly elevated levels of TMAO in AD mice result in hippocampal damage and inflammatory responses [342]. TMAO possesses the ability to traverse the blood‐brain barrier, facilitating the aggregation of Aβ and tau proteins, and modulating the expression of diverse miRNAs implicated in neurodegenerative pathways [343]. Among these, the mTOR can modulate synaptic function [344]. TMAO stimulates the activation of mTOR via the PI3K/Akt/mTOR signaling cascade [342, 345], leading to a reduction in hippocampal synaptic plasticity through the mTOR/ribosomal protein S6 kinase (P70S6K)/eukaryotic translation initiation factor 4E‐binding protein 1 (4EBP1) pathway, ultimately impacting cognitive function [344, 346].

Other metabolites that have a mechanism of action on AD but not on T2DM.

Numerous investigations have shown that the gut microbiota possesses the metabolic capability to synthesize spermidine and spermine, and conversely, dietary intake of spermidine and spermine can exert an influence on the composition of the intestinal microbiota [347, 348]. Spermidine and spermine serve as both growth factors for intestinal probiotics and regulators of mucosal cell growth, facilitating the colonization of beneficial bacteria while inhibiting harmful species, thereby preserving intestinal homeostasis. The spermidine and spermine produced by gut bacteria inhibit chronic inflammation and enhance the intestinal barrier in the colon, while also being transported into the bloodstream through the colonic epithelium. This results in significant improvements in cognitive ability and extended lifespan in murine hosts [348]. Furthermore, increased levels of spermidine and spermine may represent neuroprotective mechanisms against Aβ toxicity [349]. Autophagy is a primary mechanism of spermidine‐mediated neuroprotection. Spermidine improves mitochondrial respiration, mitochondrial membrane potential, and ATP production in cells expressing tau protein with P301L mutation (P301L tau), reduces free radical levels, enhances autophagy, and restores P301L tau‐induced mitophagy impairment [350].

### The potential comorbidity mechanisms of gut microbiota and metabolites in T2DM and AD

**FIGURE 4:**
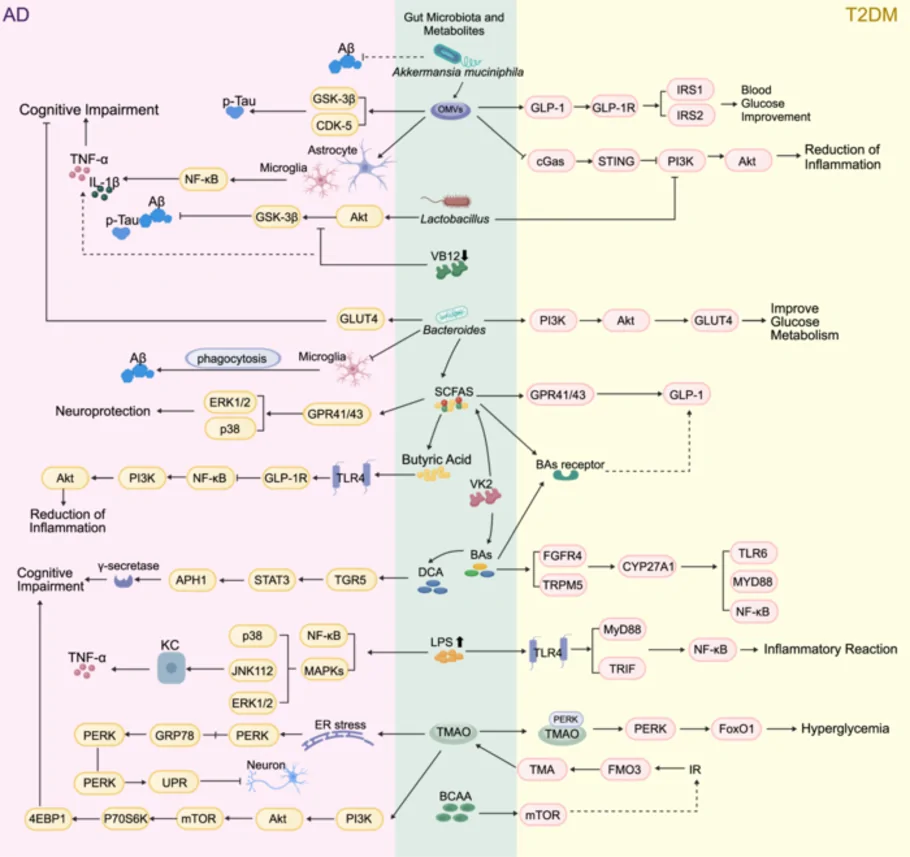
Common mechanisms of gut microbiota and metabolites in type 2 diabetes mellitus (T2DM) and Alzheimer's disease (AD). *Akkermansia muciniphila* plays a pivotal role by releasing outer membrane vesicles (OMVs) to regulate glucose and other related signaling pathways in T2DM, while in AD, it reduces amyloid‐beta (Aβ) deposition in the cerebral cortex and specific brain regions. *Lactobacillus* and *Bifidobacterium* species play a role in modulating glycogen metabolism in T2DM and mitigating gut microbiota dysbiosis, as well as associated pathological manifestations in AD, while *Bacteroides* demonstrates distinct effects in both conditions. Among metabolites, short‐chain fatty acids (SCFAs) enhance glucose homeostasis via the SCFAs‐G‐protein coupled receptor 41/43 (GPR41/43)‐glucagon‐like peptide‐1 (GLP‐1) pathway in T2DM, and activate relevant signaling pathways to exert neuroprotective effects in AD. Bile acids (BAs) exert an influence on metabolic processes and inflammatory responses in T2DM, and their metabolite, deoxycholic acid (DCA), affects brain tissue structure and glucose uptake in AD, with the potential to reverse damage by inhibiting *Bacteroidetes*. Lipopolysaccharide (LPS) in T2DM can stimulate cellular inflammatory responses through the induction of associated signaling pathway expression, thereby influencing disease progression. In AD, LPS acts as a potent neurotoxic glycolipid, capable of inducing phosphorylation of multiple kinases and nuclear factor kappa‐light‐chain‐enhancer of activated B cells (NF‐κB), stimulating neuroinflammation, and potentially promoting the secretion of tumor necrosis factor‐α by Kupffer cells. Trimethylamine N‐oxide (TMAO) promotes hyperglycemia through related signaling pathways in T2DM and influences cognitive function via the endoplasmic reticulum (ER) stress‐mediated PKR‐like endoplasmic reticulum kinase (PERK) signaling pathway in AD. Furthermore, regarding signaling pathways, the phosphoinositide 3‐kinase (PI3K)/protein kinase B (Akt) pathway is regulated by multiple factors to modulate glycogen synthesis in T2DM and is associated with Tau protein phosphorylation (p‐Tau) and cognitive impairment in AD. The Toll‐like receptor 4 (TLR4)‐ NF‐κB signaling pathway is involved in the regulation of inflammatory responses in both T2DM and AD.

By examining the mechanisms of action of gut microbiota and its metabolites on T2DM and AD, it is evident that several key proteins are involved in the common pathways underlying the pathogenesis of both diseases (Figure 4). For instance, intestinal probiotics can regulate PI3K and Akt signaling molecules to improve glycogen uptake and neuronal function in both T2DM and AD [189, 295]. The gut microbiota metabolite SCFAs can activate GPR41/43 in intestinal epithelial cells in both T2DM and AD, leading to the production of beneficial substances through various pathways [195, 306, 307, 308]. Decreasing levels of the gut microbiota metabolite LPS can activate NF‐κB via various pathways, achieving the same effect of controlling the inflammatory response in both T2DM and AD [179, 251, 252, 253, 325, 326]. The gut microbiota metabolite TMAO can cause different damages to T2DM and AD by upregulating PERK [269, 270, 337]. Furthermore, OMVs exert an influence on both T2DM and AD, yet the outcomes they induce are diametrically opposed [82, 183, 296, 297]. These findings fully demonstrate the complex interplay between T2DM and AD.

Although current research has revealed several common proteins and signaling pathways involved in the mechanisms by which gut microbiota and their metabolites impact both T2DM and AD, the exact molecular mechanisms underlying their joint regulation of these two diseases are still not fully elucidated, warranting further intensive investigation to establish a more precise foundation for personalized treatment strategies. Secondly, clinical translation encounters challenges owing to interindividual variability in microbiota composition and the intricate nature of diseases. Most studies have focused on laboratory animal experiments, and their results are often difficult to replicate in actual human populations.

### Future perspectives

Looking ahead, with the progression of high‐throughput technologies in genomics, metabolomics, and microbiomics, we foresee a more comprehensive unraveling of the specific mechanisms underlying the comorbidity of gut microbiota and its metabolites in T2DM and AD. This will provide a solid theoretical foundation for the formulation of innovative therapeutic approaches centered on gut microbiota modulation. For instance, modulating the composition and function of the gut microbiota to favor the proliferation of beneficial bacteria while suppressing the growth of harmful bacteria could emerge as a novel strategy for the prevention and treatment of T2DM and AD. Furthermore, the employment of gut microbiota metabolites, including SCFAs, LPS, and TMAO, as biomarkers can aid in the early detection of at‐risk populations for T2DM and AD, as well as in the evaluation of treatment effectiveness. Lastly, future investigations should also concentrate on elucidating the interactions between gut microbiota and other organ systems to achieve comprehensive treatment for complex comorbidities such as T2DM and AD.

## 4 CONCLUSION

This article provides an overview of the impacts and mechanisms of action exerted by gut microbiota and their metabolites on T2DM and AD. This study unveils that alterations in gut microbiota composition markedly influence the onset and progression of both diseases through the modulation of metabolic pathways, immune functions, and inflammatory responses. Key bacteria, such as *A. muciniphila* (which releases outer membrane vesicles), *Lactobacillus*, and *Bifidobacterium*, as well as their metabolites like SCFAs, BAs, LPS, and TMAO regulate T2DM and AD through complex mechanisms. These include TLR4/NF‐κB‐driven neuroinflammation, PI3K/Akt‐mediated insulin resistance, and microbial amyloid cross‐seeding, which collectively bridge the two diseases. Several signaling pathways, including GPR41/43, PI3K/Akt, TLR4/NF‐κB, and those mediated by ER stress, play pivotal roles in these processes. Future research should aim to validate these mechanistic links in animal studies and human cohorts, and develop interventions targeting the microbiota (e.g., precision probiotics, FXR agonists for bile acid modulation) to simultaneously alleviate metabolic and neurodegenerative pathologies.

## AUTHOR CONTRIBUTIONS

**Guangyi Xu**: Writing—original draft; visualization; conceptualization; investigation. **Yu An**: Project administration; writing—review and editing. **Yage Du**: Visualization; conceptualization. **Zhaoming Cao**: Visualization; formal analysis. **Jie Zheng**: Data curation. **Jingya Wang**: Data curation. **Tingyi Li**: Visualization; project administration. **Xingen Lei**: Project administration; supervision. **Yanhui Lu**: Writing—review and editing; project administration; supervision; funding acquisition; resources.

## CONFLICT OF INTEREST STATEMENT

The authors declare no conflicts of interest.

## 1 ETHICS STATEMENT

No animals or humans were involved in this study.

## Data Availability

Data sharing is not applicable to this article as no datasets were generated or analyzed during the current study. No new data and scripts were used for this review. Supplementary materials (graphical abstract, slides, videos, Chinese translated version, and update materials) may be found in the online DOI or iMeta Science http://www.imeta.science/imetaomics/.
